# Enhancement of Immune Responses by Guanosine-Based Particles in DNA Plasmid Formulations against Infectious Diseases

**DOI:** 10.1155/2019/3409371

**Published:** 2019-05-22

**Authors:** Saritza Santos, Maité Ramírez, Eric Miranda, Nelson Reyes, Osmarie Martínez, Maxier Acosta-Santiago, José M. Rivera, Miguel Otero

**Affiliations:** ^1^Department of Microbiology and Medical Zoology, University of Puerto Rico, Medical Sciences Campus, San Juan, Puerto Rico, USA; ^2^School of Science and Technology, Universidad del Este, Carolina, Puerto Rico, USA; ^3^Department of Chemistry, University of Puerto Rico, Rio Piedras Campus, San Juan, Puerto Rico, USA

## Abstract

Immunogenicity of DNA vaccines can be efficiently improved by adding adjuvants into their formulations. In this regard, the application of nano- and microparticles as vaccines adjuvants, or delivery systems, provides a powerful tool in designing modern vaccines. In the present study, we examined the role of “Supramolecular Hacky Sacks” (SHS) particles, made via the hierarchical self-assembly of a guanosine derivative, as a novel immunomodulator for DNA plasmid preparations. These plasmids code for the proteins HIV-1 Gag (pGag), the wild-type vaccinia virus Western Reserve A27 (pA27L), or a codon-optimized version of the latter (pOD1A27Lopt), which is also linked to the sequence of the outer domain-1 (OD1) from HIV-1 gp120 protein. We evaluated the enhancement of the immune responses generated by our DNA plasmid formulations in a murine model through ELISpot and ELISA assays. The SHS particles increased the frequencies of IFN-*γ*-producing cells in mice independently immunized with pGag and pA27L plasmids. Moreover, the addition of SHS to pGag and pA27L DNA plasmid formulations enhanced the production of IFN-*γ* (Th1-type) over IL-4 (Th2-type) cellular immune responses. Furthermore, pGag and pA27L plasmids formulated with SHS, triggered the production of antigen-specific IgG in mice, especially the IgG2a isotype. However, no improvement of either of those adaptive immune responses was observed in mice receiving pOD1A27Lopt+SHS. Here, we demonstrated that SHS particles have the ability to improve both arms of adaptive immunity of plasmid coding “wild-type” antigens without additional strategies to boost their immunogenicity. To the best of our knowledge, this is the first report of SHS guanosine-based particles as DNA plasmid adjuvants.

## 1. Introduction

DNA plasmid vaccines represent a simple, safe, and effective alternative for the activation of adaptive immunity. They have the capacity to present the encoded antigen through the MHCI and MHCII pathways [1]. Additionally, the antigenic protein can undergo posttranslational modifications as in a natural infection [2]. Although some have been licensed for veterinary purposes [3], they show lower immunogenicity in larger organisms, limiting their use in humans to clinical trials [4]. The use of electroporation as delivery strategy [5, 6], antigen sequence optimization [7, 8], the application of heterologous vaccine platforms [9, 10], and the inclusion of adjuvants into formulation [11, 12] are some of the multiple strategies that have been developed to overcome this limitation identified on DNA vaccines.

Adjuvants work by enhancing vaccine immunity through several mechanisms. They are classified into five categories: mineral compounds, bacterial products, oil-based emulsions, immunostimulatory complexes (ISCOMs), and liposomes. Currently, the inorganic alum-based compounds are the only FDA-approved adjuvants to be included in vaccines administered in the United States [13]. However, alum is not suitable for all vaccine formulations as its immunomodulatory properties greatly depend on the nature of the antigen and mainly stimulate humoral immunity over cell-mediated immunity [14, 15]. Therefore, the development of more efficient adjuvants and/or vaccine delivery systems to obtain high and long-lasting Th1 cell-mediated immune responses is of primary importance.

An innovative approach is the use of nanoparticles as vaccine delivery systems or immunomodulators (nanovaccinology) [16, 17]. Nanoparticles differing in size, shape, surface, and composition have been developed not only to improve antigen stability and immunogenicity, but also to allow for targeted delivery and slow release [18]. Moreover, particles sharing similar sizes with pathogens facilitate their recognition by APCs. Many nanoparticles along with traditional adjuvants have been tested in DNA vaccines [19–23].

**Figure 1:**
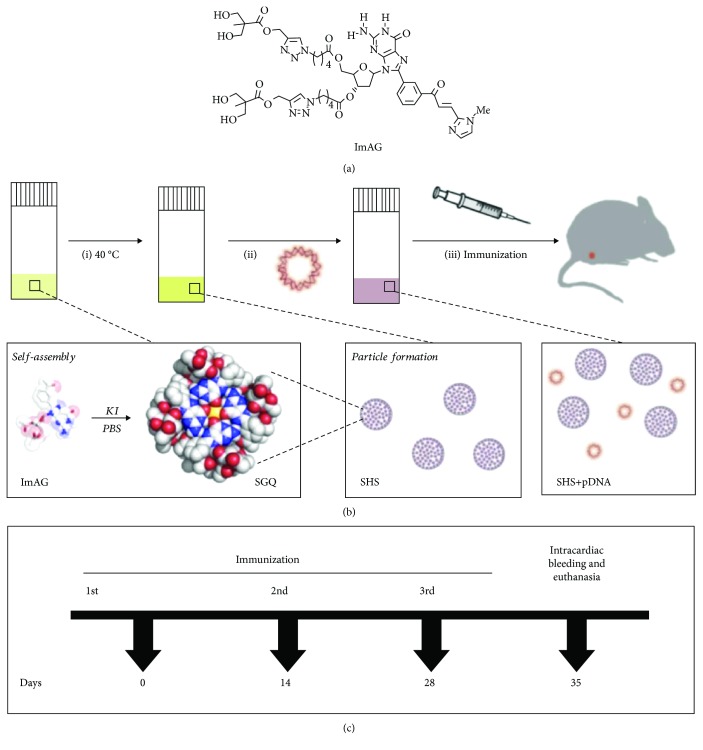
G-derivative, SHS particle preparation, and immunization schedule. (a) Structural representation of the G-derivative (ImAG) constituent of the SHS particles. (b) Schematic representation of the preparation protocol for the formulations used. (c) Each mouse was intramuscularly immunized with 100 *μ*g of the pDNAs with or without 0.1515 mM SHS in 100 *μ*L of PBS (50 *μ*L/leg) on days 0, 14, and 28. Mice were bled intracardially and euthanized by cervical dislocation on the 7th day after the third immunization, following the guidelines established by the IACUC and NIH.

Here, we report the use of Supramolecular Hacky Sacks (SHS) particles, as a novel immunomodulator for DNA plasmid formulations. SHS are the resulting structure of thermo-responsive supramolecular guanosine quadruplexes (SGQs), previously reported in 2015 [24]. Guanosine and its derivatives are known to self-assemble into planar tetrameric structures [25]. The SGQ itself is the self-assembled structure of the imidazole-8-aryl-2′-deoxyguanosine (ImAG) derivative in the presence of potassium ion K^+^ [26]. As seen in Figure 1(a), ImAG has hydrocarbon chains ending with the OH groups at the 3′ and 5′ positions of the guanine and a chalcone moiety at the C8 position composed of a phenyl group and a methylated imidazole group [27]. We decided to evaluate these particles for immunotherapeutic applications, mainly motivated by the feasible synthesis of ImAG and the simple preparation of the SHS. Furthermore, these mesoglobular structures with a size of ~1 *μ*m in a suspension-like solution can interact with a variety of cargo from small molecules (e.g., doxorubicin) to large DNA plasmids by electrostatic interactions on the SHS surface as previously described in 2016 [28]. Under scanning electron microscopy (SEM), SHS particles are visualized as organized colloidal particles with a porous gel-like architecture that can exhibit flower-shape, depending on the size and nature of the cargo (Figure S1).

In the present study, we aim to investigate the immunomodulatory properties of SHS particles in three different DNA plasmid formulations: one of them is coding for the HIV-1 Gag polyprotein (pGag) and the other two were for the wild-type (pA27L) or the codon-optimized clone (pOD1A27Lopt) of vaccinia virus Western Reserve (VVWR) A27 protein, respectively. Gag is the precursor for many structural proteins important for assembly process of HIV [29], while A27 protein is localized in the viral membrane and mediates the viral particle fusion with the cell membrane [30]. Additionally, the pOD1A27Lopt plasmid contains the sequence of the outer domain-1 (OD1) of the gp120 envelope glycoprotein from HIV-1 [31–33], to promote the internalization and presentation of antigen A27 to the immune system through recognition by DC-SIGN receptor on the surface of dendritic cells. In summary, SHS were coformulated with pGag, pA27L, or pOD1A27Lopt plasmids to immunize mice in order to evaluate their effects in adaptive immune responses through analysis of antibody and cytokine production.

## 2. Materials and Methods

### 2.1. Plasmid Design

BlueHeron Biotechnology (Bothell, WA, USA) synthesized the plasmids containing the sequences for VVWR A27L (GenBank #AAN78218.2) or HIV-1 Gag (NL4-3 clone) genes (GenBank #AAK08483.1). A27L, OD1A27Lopt, and Gag sequences were cloned into the HindIII and NotI, BamHI and NotI, and BamHI and XhoI, respectively, of the commercially available pVAX1 (Invitrogen, Grand Island, NY, USA) vector. The plasmid backbone includes a cytomegalovirus (CMV) promoter, a bovine growth hormone (BGH) polyadenylation signal, and a kanamycin resistance gene for antibiotic-based selection. Molecular modifications were also incorporated to construct in order to enhance protein expression and purification, such as the insertion of a Kozak consensus sequence, a hemagglutinin tag (HA), and an IgE leader sequence (File S1). Additionally, the pOD1A27Lopt plasmid was synthesized and optimized using the GeneOptimizer Approach by Life Technologies (Waltham, MA, USA). It contains the codon-optimized VVWR A27L and HIV-1 outer domain-1 (OD1) sequences, which are intended to serve as additional adjuvants.

### 2.2. Study Agents

#### 2.2.1. DNA Plasmid Preparation

DNA plasmids (pVAX1, pGag, pA27L, and pOD1A27Lopt) were propagated by transformation of TOP10 Chemically Competent *E. coli* cells (Invitrogen, Valencia, CA, USA). PureLink® HiPure Plasmid DNA GigaPrep Kit (Invitrogen, Carlsbad, CA, USA) was used for plasmid purification. The plasmids were resuspended in ultrapurified water and stored at -20°C until further use.

The integrity of constructs was verified by double enzymatic digestion (New England Biolabs, Ipswich, MA, USA) and DNA sequencing (Molecular Biology Core Facility, UPR: RCM-RCMI Program, San Juan, PR, USA). The agarose gel electrophoresis separation showed the expected 1.6 kbp (pGag), 0.4 kbp (pA27L), and 1.4 kbp (pOD1A27Lopt) bands (Figure S2). Assembly validation was completed using the Bioinformatics Software MacVector (MacVector Inc., Apex, NC, USA).

#### 2.2.2. SHS Particle Preparation and Formulation Protocols

G-derivative ImAG (Figure 1(a)) was synthesized and characterized as previously reported [24, 27, 28]. A solution of 0.303 mM of ImAG SGQ was warmed in a water bath at 40°C for ~3 minutes in order to promote the formation of the SHS particles and added at a final concentration of 0.1515 mM into corresponding DNA plasmid formulations (Figure 1(b)). The DNA plasmid concentration was adjusted to 1 *μ*g/*μ*L in order to prepare the formulations that were administrated to mice. Our preparations consisted of 100 *μ*g of pVAX1, pGag, pA27L, or pOD1A27Lopt in 100 *μ*L of phosphate-buffered saline (PBS) with or without 0.1515 mM of SHS particles at a ratio of 1 : 4, respectively (Table 1). The concentration of SHS used in this study (0.1515 mM) was based on preliminary results from experiments with formulations consisting of 100 *μ*g of pA27L and two different doses (0.01515 mM and 0.1515 mM) of SHS particles (Figure S3).

### 2.3. Animals and Immunization Regimen

The 4-6-week-old female BALB/c mice were purchased from Charles River (Wilmington, MA, USA) and were maintained in a pathogen-free facility following the guidelines to minimize pain and distress, established by the National Institutes of Health (Bethesda, MD, USA), as well as in our approved protocol (#9250116) of the University of Puerto Rico Institutional Animal Care and Use Committee (IACUC). Mice (*n* = 4/group) received three doses of 100 *μ*g of each DNA plasmid formulated in 100 *μ*L of PBS with or without 0.1515 mM of SHS by intramuscular needle injection in the thigh skeletal muscles following the strategy shown in Figure 1(c). Total injection volume administered to all mouse groups was 100 *μ*L (50 *μ*L/leg) among immunizations. The spleens and sera from immunized mice were collected one week after the last immunization to perform cellular and humoral immunoassays, respectively.

### 2.4. Synthetic Peptides

JPT Peptide Technologies (Berlin, Germany) designed the peptide pools (15-mer peptides overlapping 11 amino acid residues at the amino terminal) from the HIV-1 Gag consensus subtype B (123 peptides) and the VVWR A27 (25 peptides) proteins used in this study. They were used as a single peptide library pool consisting of 15 nmol (approx. 25 *μ*g) of each peptide spanning the corresponding whole antigenic protein sequence. These were diluted to a concentration of 0.5 mg/mL in RPMI for ELISpot and Quantikine ELISA analyses.

### 2.5. ELISpot Analysis

The ELISpot assay was carried out to determine the frequency of cells producing IFN-*γ*. Briefly, high-protein binding IP 96-well Multiscreen TM plates (Millipore, Bedford, MA, USA) were coated overnight at 4°C with anti-mouse IFN-*γ* capture antibody (R&D Systems, Minneapolis, MN, USA). Then, plates were washed with sterile PBS and blocked with 1% BSA. Subsequently, 2 × 10^5^ splenocytes/well were stimulated with the HIV-1 Gag (consensus subtype B) or VVWR A27 peptide mix (JPT Peptide Technologies, Berlin, Germany) overnight at 37°C, in 5% CO_2_. Culture media and concanavalin A (Con A, 1 *μ*g/mL; Sigma-Aldrich, St. Louis, MO, USA) were included as negative and positive controls, respectively. Afterwards, anti-mouse IFN-*γ*-biotinylated detection antibody was added and incubated overnight at 4°C. Plates were developed by the addition of streptavidin-alkaline phosphatase and incubation with BCIP/NBT substrate (R&D Systems, Minneapolis, MN). Finally, plates were rinsed with distilled water and air-dried overnight. Spot-forming cells (SFC) were enumerated with the ImmunoSpot software in an automated ELISpot reader system (CTL analyzers, Cleveland, OH, USA). The mean number of spots from triplicate wells was normalized for 10^6^ splenocytes. Antigen-specific frequency of IFN-*γ*-producing cells was determined subtracting the average counts of spots in negative control wells from average counts in peptide-stimulated wells.

### 2.6. Cytokine Profile

The Quantikine Mouse IFN-*γ* and IL-4 Immunoassays (R&D Systems, Minneapolis, MN, USA) were performed to measure IFN-*γ* and IL-4 levels in supernatant of mouse splenocytes, as previously described [7]. Briefly, splenocytes (2 × 10^5^ cells/well) from immunized mice were cocultured for five days (37°C, 5% CO_2_) in a 96-well flat-bottom plate with the HIV-1 Gag (consensus subtype B) or VVWR A27 peptide mix. After stimulation, supernatants were collected and frozen at -80°C until the analyses. Cytokine concentrations were determined after correlating with a standard curve.

### 2.7. Serum Antigen-Specific IgG, IgG_1_, and IgG_2a_ ELISA

One week after the last immunizations, sera from immunized mice were tested for the detection of total IgG and its isotypes, IgG1 and IgG2a, through indirect ELISA. Briefly, 96-well plates were coated overnight at 4°C with 80 *μ*g/mL of HIV-1 p24 protein (Abcam, Cambridge, MA, USA) or 1 *μ*g/mL of VVWR A27 (BEI Resources, NIAID, NIH, Manassas, VA, USA) dissolved in 0.05 M carbonate-bicarbonate buffer (pH 9.6). Then, plates were washed with PBS-T and blocked with a solution containing PBS-T, 5% FBS, and 5% dry milk for 2 hours at room temperature. Subsequently, mouse serum samples were diluted in blocking reagent as follows: 1 : 1,600 (for pA27L assays), 1 : 12,800 (for pOD1A27Lopt assays), or 1 : 25 (for pGag assays), added to corresponding plates and incubated for 2 hours at room temperature (RT). These serum dilutions were previously optimized for each antigen in our laboratory. We choose the lower ones that showed a ratio >5 between optic density (OD) from naïve and pDNA mouse sera. After a washing step, plates were incubated for 1 hour at RT with 1 : 2,500 dilutions of HRP-conjugated goat anti-mouse IgG, IgG1, or IgG2a (Jackson ImmunoResearch, West Grove, PA, USA) antibodies. Then, plates were read at wavelength 450 nm on an ELISA reader 30 minutes after rinsing and addition of OPD peroxidase substrate (Sigma-Aldrich, St. Louis, MO, USA). Reaction was stopped by addition of 50 *μ*L of 3 M HCl per well.

### 2.8. Safety Assessment

Possible adverse reactions due to administration of the formulations to female BALB/c mice were evaluated during 35 days of the study. No visible weight loss, inflammation, allergic responses, or any other undesirable effects, such as edema, were observed in mice.

### 2.9. Statistical Analysis

Statistical analyses were performed using the GraphPad Prism software (La Jolla, CA, USA) and based on three or more independent immunization studies with at least three replicates for each immunological assay done. Mean differences were calculated using the Mann–Whitney test and the two-tailed unpaired Student *t*-test to assess differences between the adjuvanted and nonadjuvanted mouse groups. Significance level criterion was set at ^∗∗∗∗^ *p* ≤ 0.0001, ^∗∗∗^ *p* ≤ 0.001, ^∗∗^ *p* ≤ 0.01, and ^∗^ *p* ≤ 0.05. Results from each experiment are expressed as mean ± standard error of the mean (SEM).

## 3. Results

### 3.1. Antigen-Specific IFN-*γ* Production

In order to evaluate the immunomodulatory properties of SHS over cell-mediated immune response, we immunized mice and determined the frequency of antigen-specific IFN-*γ*-producing splenocytes stimulated with a HIV-1 Gag (consensus subtype B) or VVWR A27 peptide pools using ELISpot assay.

**Figure 2:**
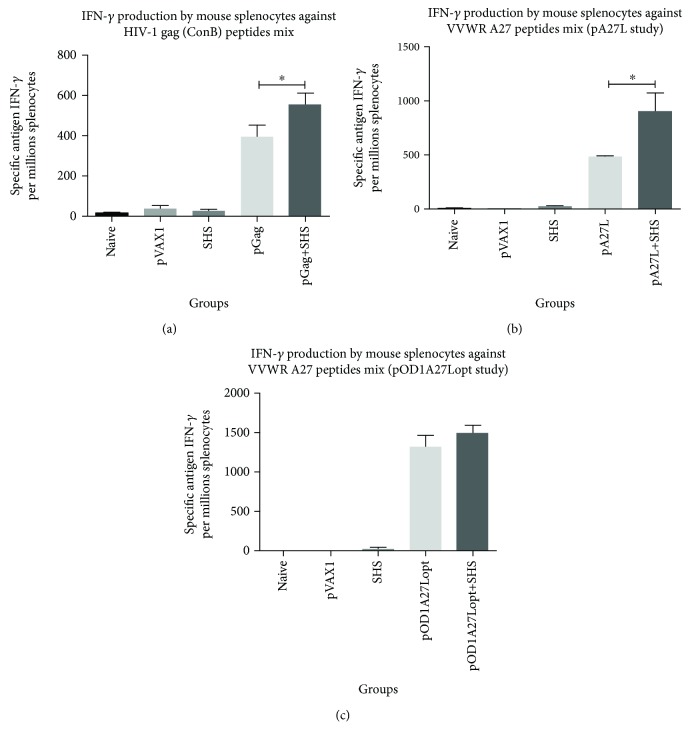
Effects of coadministration of SHS particles in antigen-specific cellular immune responses examined by ELISpot assays. The groups of female BALB/c mice (*n* = 4/group) were immunized intramuscularly, three times with a two-week interval. One week after the last immunization, cell-mediated immunity was assessed by IFN-*γ* ELISpot assays. (a) IFN-*γ* ELISpot assay of splenocytes stimulated with HIV-1 Gag (consensus subtype B) peptide mix (^∗^ *p* = 0.0260). (b) IFN-*γ* ELISpot assay of splenocytes stimulated with VVWR A27 peptide mix (pA27L study) (^∗^ *p* = 0.0151). (c) IFN-*γ* ELISpot assay of splenocytes stimulated with VVWR A27 peptide mix (pOD1A27Lopt study) (*p* = 0.2547). Data are shown as the mean ± SEM of at least three independent immunization studies with three replicates. Con A data is not shown as it goes out of scale.

We noted statistically significant 1.4- and 2.0-fold increases in spot-forming cells (SFC)/1 × 10^6^ splenocytes when SHS were coformulated with pGag or pA27L versus mice receiving DNA alone, respectively (Figures 2(a) and 2(b)). However, when SHS were added to pOD1A27Lopt formulation, we did not observe significant changes in SFC/1 × 10^6^ splenocytes producing IFN-*γ* between mice receiving adjuvanted and nonadjuvanted DNA preparations (Figure 2(c)). Moreover, administration of SHS particles alone did not considerably activate lymphocytes against Gag or A27 as measured by IFN-*γ* secretion. Therefore, these findings suggest that coadministration of SHS with pGag and pA27L plasmids improves antigen-specific cell-mediated immunity by increasing IFN-*γ* production, but failed to enhance this response in pOD1A27Lopt preparation.

### 3.2. Cytokine Detection

In order to determine which type of cell-mediated immunity (Th1 or Th2) was enhanced by addition of SHS to our DNA plasmid formulations, we compared the relative concentration levels of IFN-*γ* and IL-4 in the supernatant of splenocytes from immunized mice upon stimulation with HIV-1 Gag (consensus B) or VVWR A27L peptide mix.

**Figure 3:**
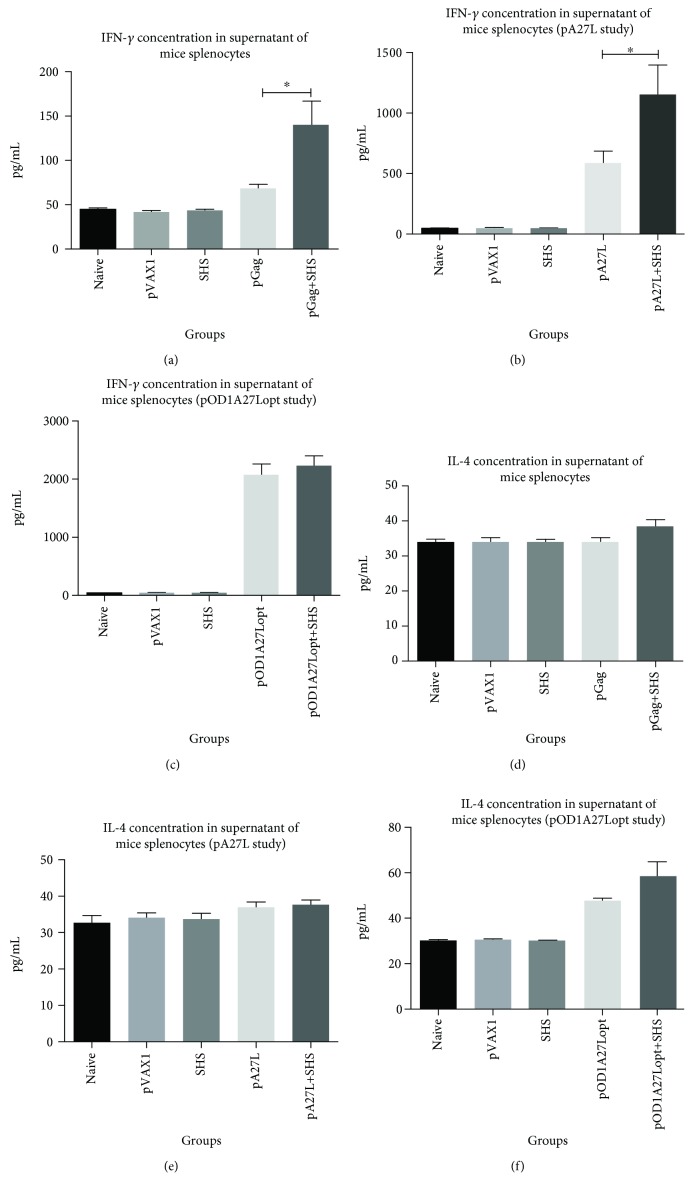
Th1 and Th2 cytokine profile as result of codelivery of pDNAs and SHS particles by Quantikine® ELISA. Seven days after last immunization, splenocytes from mouse groups (*n* = 4/group) under study were stimulated for five days with HIV-1 Gag or VVWR A27-overlapping peptide mix. Afterwards, the culture supernatants were collected to determine the IFN-*γ* and IL-4 concentration. Cytokine levels were determined by correlating with a standard curve. (a) Levels of antigen-specific produced IFN-*γ* in response to HIV-1 Gag peptide mix (^∗^ *p* = 0.0283). (b) Levels of antigen-specific produced IFN-*γ* in response to VVWR A27 peptide mix (pA27L study) (^∗^ *p* = 0.0431). (c) Levels of antigen-specific produced IFN-*γ* in response to VVWR A27 peptide mix (pOD1A27Lopt study) (*p* = 0.6784). (d) Levels of antigen-specific produced IL-4 in response to HIV-1 Gag peptide mix (*p* = 0.1515). (e) Levels of antigen-specific produced IL-4 in response to VVWR A27 peptide mix (pA27L study) (*p* = 0.5059). (f) Levels of antigen-specific produced IL-4 in response to VVWR A27 peptide mix (pOD1A27Lopt study) (*p* = 0.3874). Data are shown as the mean ± SEM of at least three independent immunization studies with three replicates.

SHS significantly enhanced IFN-*γ* production of pGag or pA27L by 2.0-fold increases when compared to corresponding pDNA alone administration (Figures 3(a) and 3(b)). However, we did not find significant improvement of IFN-*γ* production in the pOD1A27Lopt+SHS-immunized mice versus pOD1A27Lopt alone group (Figure 3(c)). Regarding IL-4 assays, we observed similar levels when animals received pDNAs with or without SHS particles formulations (Figures 3(d)–3(f)). We did not observe significant changes in both cytokines from mice that were immunized with SHS particles; hence, the observed responses in experimental groups were antigen-specific. Thus, we conclude that the incorporation of SHS as an adjuvant in pGag and pA27L DNA plasmid formulations improves Th1 cell-mediated immune responses, while further enhancement was not achieved with pOD1A27Lopt preparation.

### 3.3. Humoral Immune Responses

**Figure 4:**
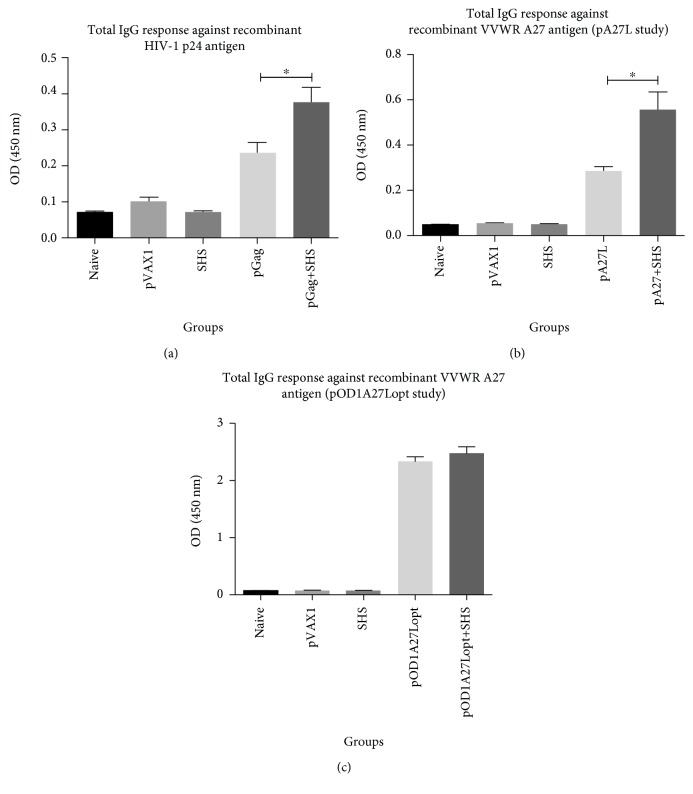
IgG antibody production due to administration of pDNAs and SHS particles as determined through ELISA. The groups of mice (*n*=4/group) were DNA-based immunized by i.m. injection, three times within a two-week interval to evaluate antibody responses. Seven days after the last immunization, we prepared serum dilutions from each group of mice and led it to react with HIV-1 Gag p24 or VVWR A27 recombinant proteins. (a) Analysis of total IgG against HIV-1 p24 protein (^∗^ *p* = 0.0379). (b) Analysis of total IgG against VVWR A27 protein (pA27L study) (^∗^ *p* = 0.0118). (c) Analysis of total IgG against VVWR A27 protein (pOD1A27Lopt study) (*p*=0.4740). Data are shown as the mean ± SEM of at least three independent immunization studies with three replicates.

We performed indirect ELISA to assess the adjuvanticity of SHS in our DNA preparations for the enhancement of antigen-specific humoral responses. Measurable IgG responses were induced by pGag, pA27L, and pOD1A27Lopt alone at dilutions ranging as follows: (a) pGag: 1 : 25-1 : 100, (b) pA27L: 1 : 400-1 : 12,800, and (c) pOD1A27Lopt: 1 : 400-1 : 204,800. However, IgG production was not detectable at a dilution of 1 : 200, 1 : 25,600, and 1 : 409,600 for pGag, pA27L, and pOD1A27Lopt, respectively. When SHS particles were added to pGag and pA27L formulations, we observed augmented IgG production. Specifically, coadministration of SHS with pGag (1 : 25 serum dilution) or pA27L (1 : 1,600 serum dilution) resulted in statistically significant 2.0-fold increases of the total IgG versus pDNA alone-immunized mouse groups, respectively (Figures 4(a) and 4(b)). Mice receiving pOD1A27Lopt+SHS (1 : 12,800 serum dilution) did not show a significant increase in IgG production versus those inoculated with pOD1A27Lopt formulation (Figure 4(c)).

**Figure 5:**
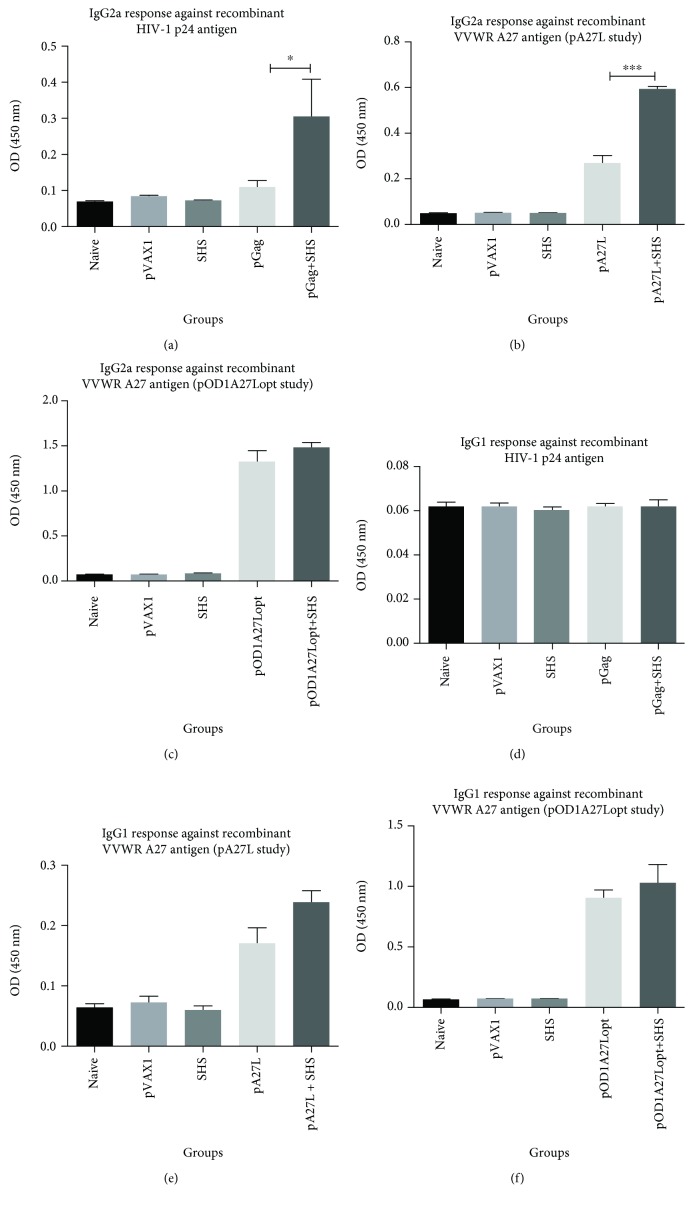
IgG isotype antibody production due to administration of pDNAs and SHS particles as determined through ELISA. The groups of mice (*n* = 4/group) were DNA-based immunized i.m., three times with a two-week interval to evaluate antibody responses. Seven days after the last immunization, we prepared serum dilutions from each group of mice and led it to react with HIV-1 Gag p24 or VVWR A27 recombinant proteins. (a) IgG2a response against HIV-1 Gag p24 protein (^∗^ *p* = 0.0260). (b) IgG2a response against VVWR A27 protein (pA27L study) (^∗∗∗^ *p* = 0.0007). (c) IgG2a response against VVWR A27 protein (pOD1A27Lopt study) (*p* = 0.3874). (d) IgG1 response against HIV-1 Gag p24 protein (*p* = 0.5565). (e) IgG1 response against VVWR A27 protein (pA27L study) (*p* = 0.1180). (f) IgG1 response against VVWR A27 protein (pOD1A27Lopt study) (*p* = 0.8983). Data are shown as the mean ± SEM of at least three independent immunization studies with three replicates.

Furthermore, analysis of IgG isotyping showed statistically significant 3.0- and 2.2-fold increases in the production of IgG2a when SHS were coformulated with pGag or pA27L versus pDNAs alone, respectively (Figures 5(a) and 5(b)). We were not able to notice further enhancement of IgG2a response in pOD1A27Lopt+SHS, when compared to the pOD1A27Lopt alone mouse groups (Figure 5(c)). Interestingly, we did not detect IgG1 production in sera from mice immunized with pGag or pGag+SHS (Figure 5(d)). Additionally, both pA27L and pOD1A27Lopt together with SHS particles showed similar IgG1 production when compared to pDNAs alone (Figures 5(e) and 5(f)). All of these responses were antigen-specific as sera from animals immunized with SHS particles alone did not show production of antibodies. These data demonstrate that pGag and pA27L combined with SHS elicited antibody responses, specifically favors the production of the isotype IgG2a. Therefore, our results are consistent with previous cell-mediated immunity experiments, which suggest that SHS enhance Th1-associated immune responses of pGag and pA27L by promoting IFN-*γ* secretion, but such enhancement was not observed for pOD1A27Lopt formulation.

## 4. Discussion

Modern vaccines, such as DNA-based formulations, often require adjuvants to improve their immunogenicity. The FDA has only approved alum-based compounds to be used in vaccines administered in the United States. Therefore, this limited availability of safe and effective immunomodulators has increased the interest to develop more efficient adjuvants and/or vaccine delivery systems, such as nanoparticles, to obtain long-lasting memory and robust cellular immune responses. In order to contribute to these efforts, we evidenced the ability of the novel SHS particles coformulated with DNA plasmid coding for HIV-1 Gag or VVWR A27 proteins, to enhance immune responses.

Although the exact mechanism of action of the SHS is still unknown, we hypothesize it might involve activation of the Toll-like receptor 7 (TLR7), which is consistent with the Th1 immune response observed in this study and with the fact that the SHS particles are made from a guanosine derivative. Other C8-substituted G-derivatives like 7-allyl-8-oxoguanosine (loxoribine) and 7-thia-8-oxoguanosine (TOG) have been known for a long time to have immunostimulatory activity [34–37], which was eventually determined to be a result of TLR7 activation [38]. However, further studies are required to elucidate the detailed molecular mechanism behind the immunological activity of SHS particles.

To test SHS particles as a novel approach to enhance cell-mediated immunity, we measured the frequency of antigen-specific IFN-*γ*-producing cells. IFN-*γ* is mainly associated with the Th1 cell-mediated immunity, which is needed to eradicate intracellular pathogens and tumors [39, 40], while IL-4 is the key orchestrator of the Th2 immune responses that are effective against extracellular microbes [41]. Our DNA preparations pGag and pA27L, coformulated with SHS, were able to increase the frequency of IFN-*γ*-producing cells in immunized mice as shown by ELISpot assay. When mice received our pDNA's formulations without SHS particles, lymphocytes showed a mixed induction of Th1 (IFN-*γ*)/Th2 (IL-4) immune responses with a moderate shift to promote a Th1 phenotype as seen in Quantikine® ELISA experiments. However, when SHS were incorporated to pGag and pA27L, we observed a notorious shift towards a Th1 immune response. This was not the case for the pOD1A27Lopt construct, in which we even noted a slightly decrease in IFN-*γ*/IL-4 ratio. This would not be the first time that an adjuvant is reported to induce a balanced mix of Th1 and Th2 immune responses, as recently, the saponin-based adjuvant G3 showed a similar behavior in BALB/c mice [42]. After observing enhancement of Th1 immune responses elicited by pGag and pA27L by adding SHS to formulations, we analyzed if SHS increased the amount of cytokine produced per splenocyte. We found a 1.5-fold increase in IFN-*γ* secreted per splenocytes when SHS were added only to pGag preparation; pA27L and pOD1A27Lopt formulations did not show significant changes. Overall, these data demonstrate the role of SHS enhancing Th1 cell-mediated immunity in pGag and pA27L, but not in pOD1A27Lopt formulation possibly due to the nature of the construct, as we will discuss later.

Cell proliferation is required to expand the repertoire of activated antigen-specific lymphocytes able to fight infections with pathogens [43]. IL-2 is an autocrine cytokine responsible for proliferation and cytokine production (IFN-*γ*, IL-4) in immune cells [44]. Although we were not able to perform cell proliferation studies, our IFN-*γ* ELISpot results allow us to speculate that SHS particles could promote the production of IL-2, leading to a higher proliferative activity on lymphocytes from the mouse spleens receiving pGag and pA27L immunizations, but not pOD1A27Lopt. However, the role of SHS particles in this immunological process must be addressed in the future.

Furthermore, we evaluated the ability of our SHS-adjuvanted pDNAs to trigger antibody production. Several studies have established the essential role of IgG antibodies for protection against both HIV and poxviruses [45, 46]. This antibody is the most abundant in serum and exerts antiviral activity by opsonizing or neutralizing viral particles and by complement-mediated lysis [47]. Additionally, it is well known that IFN-*γ* promotes the expression of IgG2a in mice, while inhibiting IgG1 production; thus, IgG2a isotype is associated with Th1 immune responses [48–50]. Although, we found low levels (OD) of antibodies in pGag experiments, this was not surprising as proteins coded by Gag gene have been widely described as antigens responsible for triggering mainly CD8^+^ T cells immune responses [51]. Even though, we still found a statistically significant improvement of IgG antibodies, specifically IgG2a in mice that received pGag+SHS formulation versus those that received pGag alone. Moreover, when pGag was administered alone, the response ratio of IgG2a to IgG1 was 1.6, but when SHS particles were added to formulation, this ratio increased to 5.0, supporting our hypothesis that SHS could boost Th1-adaptive immune responses of pGag preparation. SHS were not only able to improve T cell immunity of pA27L DNA plasmid, but we also observed an increase in total IgG production between the adjuvanted and nonadjuvanted DNA-immunized mouse groups. Furthermore, when mice received pA27L alone, the response ratio of IgG2a to IgG1 was 1.5, but when SHS particles were added to formulation, this ratio increases to 2.6, showing the polarization of humoral immune response towards a Th1-type in our SHS-adjuvanted pA27L preparation. However, as seen previously in cell-mediated immune experiments, the immune-enhancing effects of SHS particles in humoral studies were not noticeable in pOD1A27Lopt preparation. Indeed, the elicited change in response ratio of IgG2a to IgG1 between pOD1A27L and pOD1A27Lopt+SHS formulation was minimal. Therefore, SHS improves the antigen-specific humoral responses, especially those associated with the Th1 immunity only in mice receiving plasmids containing “wild antigens” without any other enhancers (e.g., codon optimization and OD1 sequence).

SHS particles were not able to improve further the immune responses elicited by our DNA construct pOD1A27Lopt possibly due that the pOD1A27Lopt+SHS formulation comprises additional molecular strategies for the enhancement of immunity, such as codon optimization and the OD1 sequence. Previously, we demonstrated the enhancement of Th1 immune responses in a murine model by optimizing the sequence of VVWR A27 gene (pA27Lopt) to improve gene stability and antigen expression [7]. Moreover, those immune responses were able to reduce viral replication and dissemination in mice challenged with vaccinia virus [52]. In the present study, we were the pioneers to report the potential immunomodulatory activity of linking the OD1 sequence from gp120 protein to pA27Lopt constructs. As we previously noted an improvement of pA27L Th1 immune responses, as well as protective efficacy to viral infection after codon optimization, we would expect an enhanced protection against vaccinia virus when inclusion of OD1 sequence and codon optimization strategies are implemented in our construct pOD1A27Lopt. However, robust studies behind its immunogenicity should be performed. The rationale to include OD1 to our construct was based on the fact that C-type lectin receptors in dendritic cells, such as DC-SIGN, recognize highly mannosylated proteins [53, 54]. As OD1 is highly mannosylated, it could facilitate the recognition of the expressed A27 antigen by DCs, leading to its rapid internalization, process, and presentation to T cells, which is the primary goal of modern vaccine adjuvant design.

Routes of vaccine administration and immunization strategies, as well as the adjuvants used in formulations, could influence the elicited immune responses against antigen(s) of interest [55]. The results of the pOD1A27Lopt study possibly indicate that there is a limit on the enhancement of immune responses against several immunogens, and certain levels of cellular and/or antibody responses that can be reached by different means could be difficult or impossible to exceed. Here, at this point of the study, we cannot establish which strategy (codon optimization/OD1 or SHS particles) is a better candidate to improve immunogenicity of DNA plasmids, as we only tested codon optimization and OD1 fusion with A27 antigen, but definitely it would be interesting to compare both strategies with Gag antigen to get a better picture in the future. Therefore, when considering SHS particles as an adjuvant for a vaccine candidate, it will be imperative to analyze the antigenic features of the immunogen and if other strategies are more feasible (e.g., sequence optimization) prior to designing the immunization scheme.

The application of SHS particles as an adjuvant in DNA plasmid coding for HIV-1 Gag or VVWR A27L antigens was investigated in this report. Coformulation of SHS with DNA plasmid coding “wild-type” antigens efficiently increased antigen-specific humoral and cell-mediated immunity, especially those associated with Th1 immune responses. Addition of OD1 sequence and codon optimization of A27 gene sequence seem to be enough to improve the immunogenicity of the pOD1A27Lopt formulation as SHS particles failed to improve it further. However, additional studies should be performed to evidence whether these SHS particles can promote activation of innate, B and T immune cells that could be related to the adaptive immune responses reported here and if these responses are enough to provide protection against viral infections. Our data supports the use of SHS particles as a novel adjuvant for the development of safer and effective DNA vaccines against infectious diseases, and provides a proof of concept for future testing SHS particles against other targets. Since the SHS particles are made from small-molecule guanosine derivatives, they offer the potential of combining the best attributes of nanovaccines with those of small-molecule immunomodulators [56, 57]. Furthermore, the fact that the SHS particles can encapsulate other biomedically relevant molecules like proteins and polysaccharides [28], expands the potential of this work to develop more elaborated vaccine formulations and related immunotherapies.

## Figures and Tables

**Table 1:** Mouse groups for the immunization studies.

Mouse groups	DNA plasmid (*μ*g)	SHS particles (mM)
Naïve	0	0
pVAX1	100	0
SHS	0	0.1515
pGag	100	0
pGag+SHS	100	0.1515
pA27L	100	0
pA27L+SHS	100	0.1515
pOD1A27Lopt	100	0
pOD1A27Lopt+SHS	100	0.1515

The dosages of injections for experimental groups (pDNAs alone or pDNAs+SHS) were as follows: 50 *μ*L per leg of pDNA preparation (1 *μ*g/*μ*L of pDNA in 100 *μ*L PBS) or 50 *μ*L per leg of pDNA+SHS preparation (1 *μ*g/*μ*L of pDNA + 0.1515 mM of SHS in 100 *μ*L PBS). Control mouse groups included those to determine basal responses (naïve) and those receiving 50 *μ*L per leg of backbone plasmid (1 *μ*g/*μ*L pVAX1 in 100 *μ*L PBS) and 50 *μ*L per leg of SHS particles alone (0.1515 mM SHS in 100 *μ*L PBS) to demonstrate nonantigen-specific immune responses.

## Data Availability

All data supporting the findings of this study, including its supplementary information files, are available from the corresponding author upon reasonable request.

## Abbreviations

8ArG: 8-Aryl-2′-deoxyguanosine
APCs: Antigen-presenting cells
BCIP: 5-Bromo-4-chloro-3-indolyl phosphate
BGH: Bovine growth hormone
BSA: Bovine serum albumin
CMV: Cytomegalovirus
Con A: Concanavalin A
DC-SIGN: Dendritic cell-specific intercellular adhesion molecule 3-grabbing nonintegrin
DNA: Deoxyribonucleic acid
ELISA: Enzyme-linked immunosorbent assay
ELISpot: Enzyme-linked immunospot
FBS: Fetal bovine serum
FDA: Food and Drug Administration
HA: Hemagglutinin
HCl: Hydrochloric acid
HIV-1: Human immunodeficiency virus-1
HRP: Horseradish peroxidase
i.m.: Intramuscular
IFN-*γ*: Interferon gamma
IgE: Immunoglobulin E
IgG: Immunoglobulin G
IgG1: Immunoglobulin 1
IgG2a: Immunoglobulin 2a
IL-4: Interleukin 4
ImAG: Imidazole-8-aryl-2′-deoxyguanosine
ISCOMs: Immunostimulatory complexes
MHCI: Major histocompatibility complex class I
MHCII: Major histocompatibility complex class II
NBT: Nitro blue tetrazolium
OD1: Outer domain-1
OH: Oxygen hydroxide
OPD: *o*-Phenylenediamine dihydrochloride
pA27L: A27L coding DNA plasmid
PBS-T: Phosphate-buffered saline Tween 20
PBS: Phosphate-buffered saline
pDNA: Plasmid DNA
pGag: Gag coding DNA plasmid
PLGA: Poly(lactic-co-glycolic acid)
pOD1A27Lopt: OD1-A27L-optimized coding DNA plasmid
RT: Room temperature
SEM: Standard error of the mean
SFCs: Spot-forming cells
SGQs: Supramolecular guanosine quadruplexes
SHS: Supramolecular hacky sacks
Th1: Type 1 T helper
Th2: Type 2 T helper
TLR7: Toll-like receptor 7
VVWR: Vaccinia virus Western Reserve.

## Supplementary Materials

Supplementary 1 File S1: Antigen DNA sequence constructs.

Supplementary 2 Figure S1. Physical structures of SHS particles. Depending on the size and nature of the cargo molecules, SHS particles can change from a colloidal gel-like interior to a flower-shaped structure (e.g., spikes-small guests- and wide petals-large guests-). Source: Negrón LM, Díaz TL, Ortiz-Quiles EO, Dieppa-Matos D, Madera-Soto B, and Rivera JM. Organic nanoflowers from a wide variety of molecules templated by a hierarchical supramolecular scaffold. Langmuir. 2016;32: 2283–2290.

Supplementary 3 Figure S2: Enzymatic digestion of DNA plasmids. DNA electrophoresis image showing the plasmids pGag, pA27L, and pOD1A27Lopt, enzymatically digested with BamHI-XhoI, HindII-NotI, and BamHI-NotI, respectively. Bands of 1.6 kbp, 0.4 kbp, and 1.4 kbp corresponding to pGag, pA27L, and pOD1A27Lopt gene inserts are highlighted with arrows, respectively.

Supplementary 4 Figure S3: Dose-response test of SHS particles in pDNA formulation. Female BALB/c mice (*n* = 4/group) were immunized intramuscularly, three times within a two-week interval. One week after the last immunization, cell-mediated immunity was assessed by an IFN-*γ* ELISpot assay of splenocytes stimulated with VVWR A27 peptide mix (^∗∗^ *p* = 0.0018). Formulations consisted of 100 *μ*g of the pA27L mixed with SHS (0.01515 mM or 0.1515 mM) in 100 *μ*L of PBS that were administered to mice in 50 *μ*L/leg on days 0, 14, and 28. The mouse control groups consisted of naïve, empty vector control (100 *μ*g of pVAX1 in 100 *μ*L PBS) and the adjuvant control (0.1515 mM SHS in 100 *μ*L PBS). Data are shown as the mean ± SEM of at least three independent immunization studies with three replicates. Con A data is not shown as it goes out of scale.
