# Supplementary material for: Enhancement of Immune Responses by Guanosine-Based Particles in DNA Plasmid Formulations against Infectious Diseases
Source: J Immunol Res. 2019 May 22;2019:3409371. doi: 10.1155/2019/3409371 (PMC6556318; doi:10.1155/2019/3409371)
Supplement: Supplementary 1 — File S1: Antigen DNA sequence constructs. [file 3409371.f1.pdf]

A) pGag DNA Insert Sequence

GGATCCGCCGCCACCATGGATTGGACTTGGATCTTATTTTTAGTTGCTGCTGCTACTAGAGTTCATTCTGGTGCGAGAGCGTCGGTATTAAGCGGGGGAGAATTAGATAAATGGGAAAAAATTCG
GTTAAGGCCAGGGGGAAAGAAACAATATAAACTAAAACATATAGTATGGGCAAGCAGGGAGCTAGAACGATTCGCAGTTAATCCTGGCCTTTTAGAGACATCAGAAGGCTGTAGACAAATACTGG
GACAGCTACAACCATCCCTTCAGACAGGATCAGAAGAACTTAGATCATTATATAATACAATAGCAGTCCTCTATTGTGTGCATCAAAGGATAGATGTAAAAGACACCAAGGAAGCCTTAGATAAGATA
GAGGAAGAGCAAAACAAAAGTAAGAAAAAGGCACAGCAAGCAGCAGCTGACACAGGAAACAACAGCCAGGTCAGCCAAAATTACCCTATAGTGCAGAACCTCCAGGGGCAAATGGTACATCAG
GCCATATCACCTAGAACTTTAAATGCATGGGTAAAAGTAGTAGAAGAGAAGGCTTTCAGCCCAGAAGTAATACCCATGTTTTCAGCATTATCAGAAGGAGCCACCCCACAAGATTTAAATACCATGC
TAAACACAGTGGGGGGACATCAAGCAGCCATGCAAATGTTAAAAGAGACCATCAATGAGGAAGCTGCAGAATGGGATAGATTGCATCCAGTGCATGCAGGGCCTATTGCACCAGGCCAGATGAG
AGAACCAAGGGGAAGTGACATAGCAGGAACTACTAGTACCCTTCAGGAACAAATAGGATGGATGACACATAATCCACCTATCCCAGTAGGAGAAATCTATAAAAGATGGATAATCCTGGGATTAAAT
AAAATAGTAAGAATGTATAGCCCTACCAGCATTCTGGACATAAGACAAGGACCAAAGGAACCCTTTAGAGACTATGTAGACCGATTCTATAAAACTCTAAGAGCCGAGCAAGCTTCACAAGAGGTA
AAAAATTGGATGACAGAAACCTTGTTGGTCCAAAATGCGAACCCAGATTGTAAGACTATTTTAAAAGCATTGGGACCAGGAGCGACACTAGAAGAAATGATGACAGCATGTCAGGGAGTGGGGG
GACCCGGCCATAAAGCAAGAGTTTTGGCTGAAGCAATGAGCCAAGTAACAAATCCAGCTACCATAATGATACAGAAAGGCAATTTTAGGAACCAAAGAAAGACTGTTAAGTGTTTCAATTGTGGCA
AAGAAGGGCACATAGCCAAAAATTGCAGGGCCCCTAGGAAAAAGGGCTGTTGGAAATGTGGAAAGGAAGGACACCAAATGAAAGATTGTACTGAGAGACAGGCTAATTTTTTAGGGAAGATCTG
GCCTTCCCACAAGGGAAGGCCAGGGAATTTTCTTCAGAGCAGACCAGAGCCAACAGCCCCACCAGAAGAGAGCTTCAGGTTTGGGGAAGAGACAACAACTCCCTCTCAGAAGCAGGAGCCGA
TAGACAAGGAACTGTATCCTTTAGCTTCCCTCAGATCACTCTTTGGCAGCGACCCCTCGTCACAATACCCATACGACGTCCCAGACTACGCTTAACTCGAG

B) pA27L DNA Insert Sequence

AAGCTTGCCGCCACCATGGACTGGACCTGGATCCTGTTCCTGGTGGCCGCTGCCACAAGAGTGCACAGCGACGGAACTCTTTTCCCCGGAGATGACGATCTTGCAATTCCAGCAACTGAATTT
TTTTCTACAAAGGCTGCTAAAAAGCCAGAGGCTAAACGCGAAGCAATTGTTAAAGCCGATGAAGACGACAATGAGGAAACTCTCAAACAACGGCTAACTAATTTGGAAAAAAAGATTACTAATGTA
ACAACAAAGTTTGAACAAATAGAAAAGTGTTGTAAACGCAACGATGAAGTTCTATTTAGGTTGGAAAATCACGCTGAAACTCTAAGAGCGGCTATGATATCTCTGGCTAAAAAGATTGATGTTCAGA
CTGGACGGCGCCCATATGAGTACCCATACGACGTCCCAGACTACGCTTAAGCGGCCGC

C) pOD1A27Lopt DNA Insert Sequence

GGATCCGCCGCCACCATGGATTGGACTTGGATCTTATTTTTAGTTGCTGCTGCTACTAGAGTTCATTCTAATGGCAGTCTAGCAGAAGAAGAGATAGTAATTAGATCTGAAAATTTCACAAACAAAT
GGCAGTCTAGCAGAAGAAGAGATAGTAATTAGATCTGAAAATTTCACAAACAATGCTAAAACTATAATAGTACAGCTGAACGAATCTGTAGTAATTAATTGTACAAGACCCAACAACAATACAAGAAA
AAGTATAAATATAGGACCAGGGAGAGCATTGTATACAACAGGAGAAATAATAGGAGATATAAGACAAGCACATTGTAACCTTAGTAAAACACAATGGGAAAACACTTTAGAACAGATAGCTATAAAATT
AAAAGAACAATTTGGGAATAATAAAACAATAATCTTTAATCCATCCTCAGGAGGGGACCCAGAAATTGTAACACACAGTTTTAATTGTGGAGGGGAATTTTTCTACTGTAATTCAACACAACTGTTTA
CTTGGAATGATACTAGAAAGTTAAATAACACTGGAAGAAATATCACACTCCCATGTAGAATAAAACAAATTATAAATATGTGGCAGGAAGTAGGAAAAGCAATGTATGCCCCTCCCATCAGAGGACAA
ATTAGATGTTCATCAAATATTACAGGGCTGCTATTAACAAGAGATGGTGGTAAGGACACGAACGGGACTGAGATCTTCAGACCTGGAGGAGGAGATATGAGGGACAATTGGAGAAGTGGCGCCG
GCGCCGGCGCCGACGGAACTCTTTTCCCCGGAGATGACGATCTTGCAATTCCAGCAACTGAATTTTTTTCTACAAAGGCTGCTAAAAAGCCAGAGGCTAAACGCGAAGCAATTGTTAAAGCCGA
TGAAGACGACAATGAGGAAACTCTCAAACAACGGCTAACTAATTTGGAAAAAAAGATTACTAATGTAACAACAAAGTTTGAACAAATAGAAAAGTGTTGTAAACGCAACGATGAAGTTCTATTTAGG
TTGGAAAATCACGCTGAAACTCTAAGAGCGGCTATGATATCTCTGGCTAAAAAGATTGATGTTCAGACTGGACGGCGCCCATATGAGTACCCATACGACGTCCCAGACTACGCTTAAGCGGCCGC

S1 File. Antigen DNA Sequence Constructs A) DNA sequence of pGag. Green and Blue nitrogen bases correspond to BamHI and XhoI restriction sites, respectively. Kozak sequence is represented by purple color, while orange is for IgE leader sequence. Black and pink colors were assigned to Gag and HA tag sequences, respectively. **B) DNA sequence of pA27L.** Green and Blue nitrogen bases correspond to HindIII and NotI restriction sites, respectively. Kozak sequence is represented by purple color, while orange is for IgE leader sequence. Black and pink colors were assigned to A27L and HA tag sequences, respectively. **C) DNA sequence of pOD1A27Lopt.** Green and Blue nitrogen bases correspond to BamHI and NotI restriction sites, respectively. Kozak sequence is represented by purple color, while orange is for IgE leader sequence. Black and pink colors were assigned to OD1 and HA tag sequences, respectively. A27Lopt sequence is gray-highlighted.
