# Supplementary figures and images for: Enhancement of Immune Responses by Guanosine-Based Particles in DNA Plasmid Formulations against Infectious Diseases
Source: J Immunol Res. 2019 May 22;2019:3409371. doi: 10.1155/2019/3409371 (PMC6556318; doi:10.1155/2019/3409371)

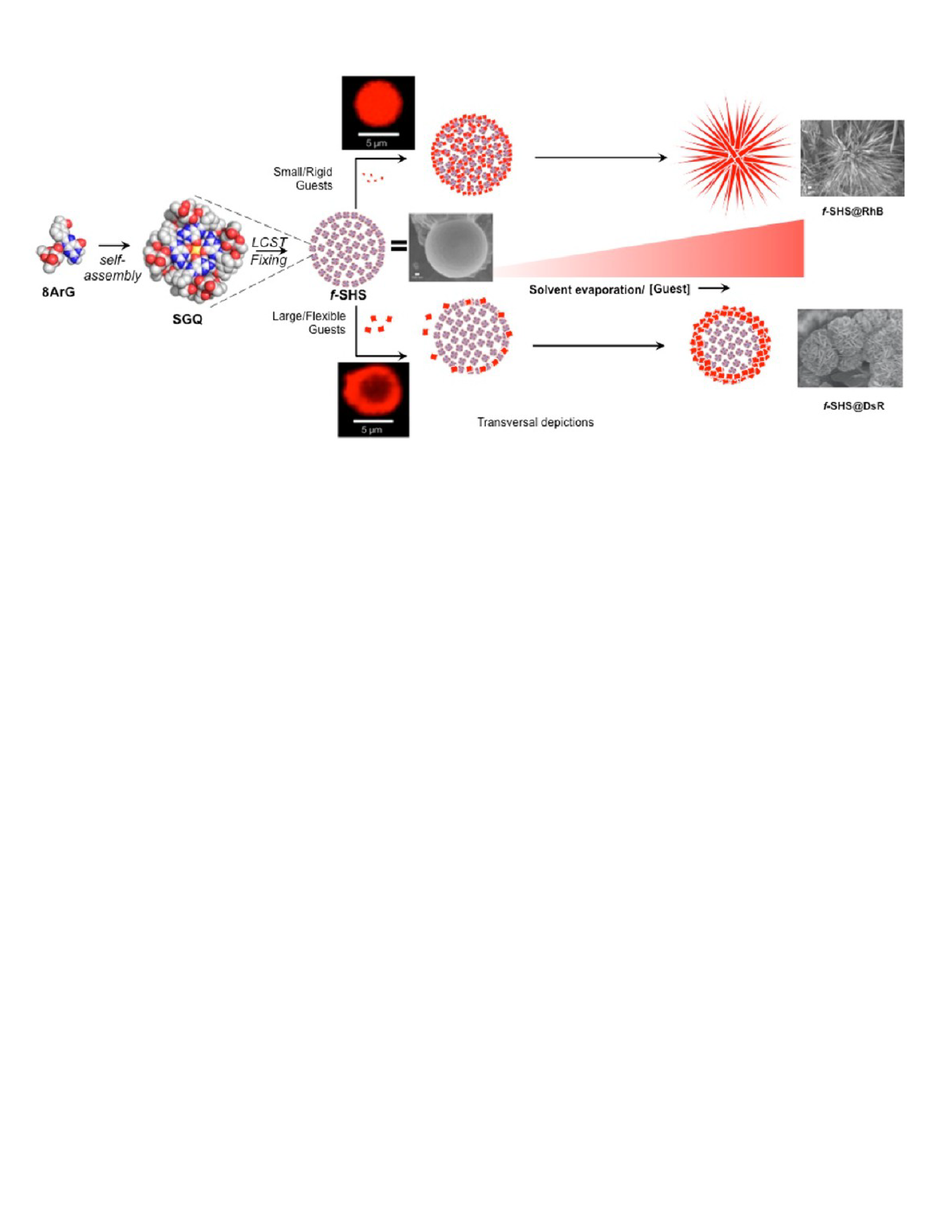

Supplement: Supplementary 2 — Figure S1. Physical structures of SHS particles. Depending on the size and nature of the cargo molecules, SHS particles can change from a colloidal gel-like interior to a flower-shaped structure (e.g., spikes-small guests- and wide petals-large guests-). Source: Negrón LM, Díaz TL, Ortiz-Quiles EO, Dieppa-Matos D, Madera-Soto B, and Rivera JM. Organic nanoflowers from a wide variety of molecules templated by a hierarchical supramolecular scaffold. Langmuir. 2016;32: 2283–2290. [file 3409371.f2.tiff]

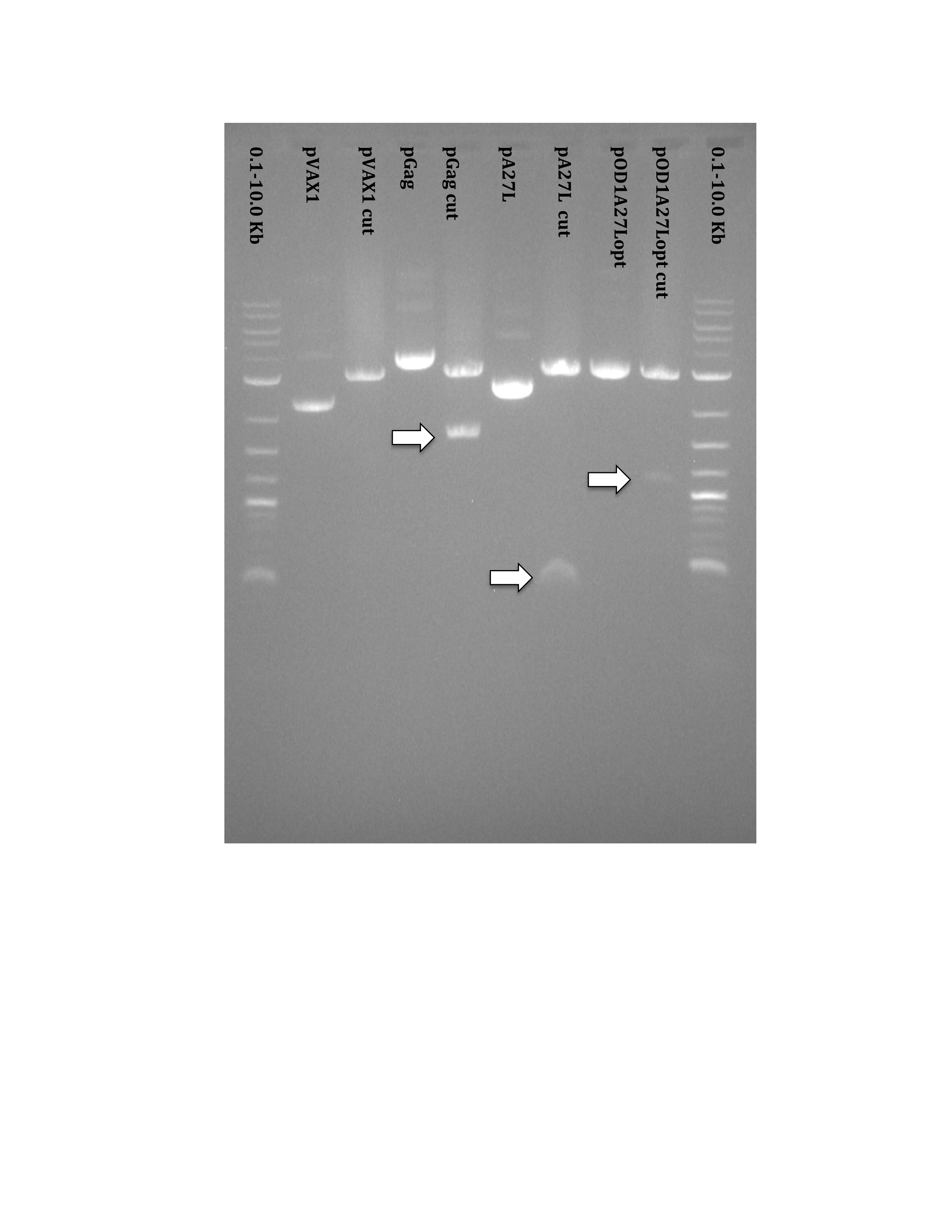

Supplement: Supplementary 3 — Figure S2: Enzymatic digestion of DNA plasmids. DNA electrophoresis image showing the plasmids pGag, pA27L, and pOD1A27Lopt, enzymatically digested with BamHI-XhoI, HindII-NotI, and BamHI-NotI, respectively. Bands of 1.6 kbp, 0.4 kbp, and 1.4 kbp corresponding to pGag, pA27L, and pOD1A27Lopt gene inserts are highlighted with arrows, respectively. [file 3409371.f3.tiff]

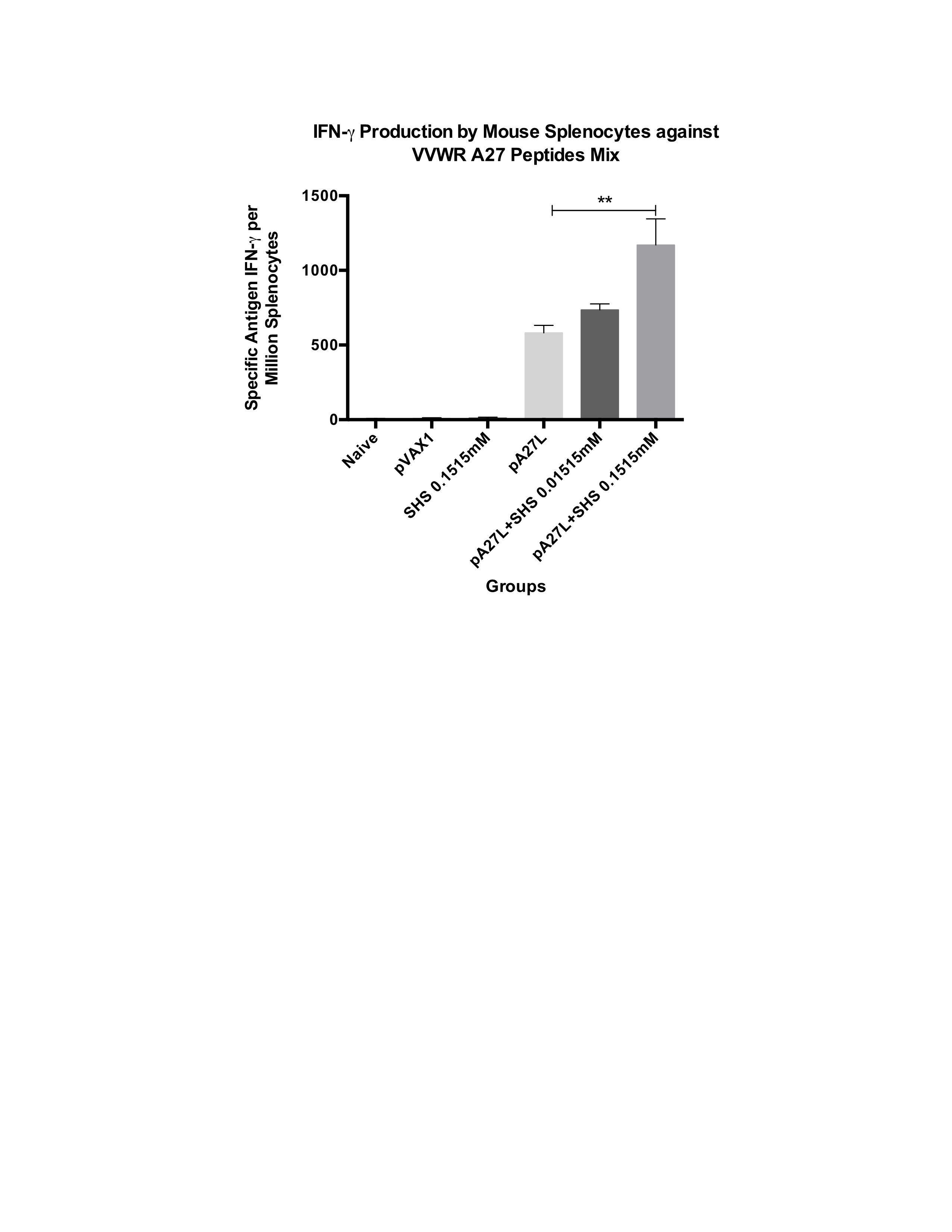

Supplement: Supplementary 4 — Figure S3: Dose-response test of SHS particles in pDNA formulation. Female BALB/c mice (n = 4/group) were immunized intramuscularly, three times within a two-week interval. One week after the last immunization, cell-mediated immunity was assessed by an IFN-γ ELISpot assay of splenocytes stimulated with VVWR A27 peptide mix (∗∗ p = 0.0018). Formulations consisted of 100 μg of the pA27L mixed with SHS (0.01515 mM or 0.1515 mM) in 100 μL of PBS that were administered to mice in 50 μL/leg on days 0, 14, and 28. The mouse control groups consisted of naïve, empty vector control (100 μg of pVAX1 in 100 μL PBS) and the adjuvant control (0.1515 mM SHS in 100 μL PBS). Data are shown as the mean ± SEM of at least three independent immunization studies with three replicates. Con A data is not shown as it goes out of scale. [file 3409371.f4.tiff]
